# Polydimethylsiloxane-Based Quantum Dot Color Conversion Layers for QD-OLED Applications

**DOI:** 10.3390/mi17050505

**Published:** 2026-04-22

**Authors:** Sang-Uk Byun, Su-Been Lee, Seo-Young Kim, Yu-Lim Seok, Gun Park, Dae-Gyu Moon

**Affiliations:** Department of Electronic Materials, Device, and Equipment Engineering, Soonchunhyang University, Asan-si 31538, Chungcheongnam-do, Republic of Korea

**Keywords:** quantum dot, color conversion layer, PDMS, blue leakage, color conversion efficiency, output/input efficiency

## Abstract

Quantum dot (QD)-based color conversion layers are key components in QD-OLED displays because they can provide high color purity and simplified pixel architectures by converting blue emission from OLEDs into red or green light. The performance of the color conversion layer strongly depends on the blue light absorption, blue leakage, and overall emission efficiency of the display. We fabricated the color conversion layers using a thermally curable polydimethylsiloxane (PDMS) matrix, and their color conversion characteristics were systematically compared with those of QD-only layers. In the QD-only layers, the intensity of the converted green emission increased with increasing QD concentration due to enhanced absorption of blue light emitted from the OLED. However, a large fraction of blue light was transmitted through the layer without being absorbed by the QDs, resulting in a significant blue leakage and a relatively low output/input efficiency below 10%. In contrast, PDMS-based QD color conversion layers exhibited substantially improved color conversion characteristics. By varying the QD concentration and controlling the layer thickness, blue leakage was significantly suppressed and the green emission intensity increased. The maximum color conversion efficiency of 30.0% was obtained at a QD concentration of 8.3 wt% with a layer thickness of 35.9 µm.

## 1. Introduction

Quantum dots (QDs) are nanoscale semiconductor particles that exhibit discrete energy levels and size-dependent optical properties due to quantum confinement effects [1,2,3]. QDs have been extensively studied during past decades because of their superior characteristics such as size-tunable wavelengths, high photoluminescence quantum yield, and narrow emission bandwidth in display applications [4,5,6,7,8,9]. In particular, QDs have attracted much attention in recent years as color conversion materials for the blue organic light-emitting devices (OLEDs), since they can provide high-quality displays by combining the advantages of OLEDs, such as self-emission and high contrast ratio, with the excellent characteristics of QDs, including high color purity and spectral tunability [10,11,12]. In the QD-OLEDs, the blue OLED serves as the excitation source and the QDs act as the color conversion layer. QDs absorb the blue light emitted from OLED and re-emit red or green light from QDs. Since red and green pixels originate from a single blue OLED, there is no need to use a fine metal mask for sub-pixel formation, offering a simpler pixel patterning process compared to RGB OLED arrays [7]. QD-OLEDs possess all the excellent characteristics of OLEDs, such as a high contrast ratio, fast response time, thin form factor, and mechanical flexibility [13,14,15]. In addition, QD-OLEDs provide better color mixing and a wider color gamut compared to conventional OLEDs due to symmetric and sharp emission characteristics of QDs [16,17,18].

In QD-OLEDs, QDs are integrated into a stable and efficient color conversion layer that is optically transparent, mechanically robust, and compatible with the OLED substrate. One of the key issues in fabricating color conversion layers is color conversion efficiency, which determines how effectively blue light is converted into green and red light. QDs exhibit superior color conversion efficiency compared to traditional phosphors due to their high quantum yield and narrow emission spectra [7]. This efficiency directly impacts the overall brightness of displays. The most common approach for fabricating a color conversion layer is to formulate QD ink by dispersing QDs in polymer solutions and forming a thin film using a solution coating method [19,20,21,22,23,24,25,26]. Among the polymer candidates, UV-curable resins have been widely used due to their simple processing, patterning capabilities, and fast curing time [21,22,23,24,25,26]. Photopolymer resin-based QD inks allow for layer-by-layer printing, making it easy to control the thickness [26]. However, UV-curable QD–polymer composites face several limitations when applied to QD-OLEDs. Prolonged exposure to UV light can degrade both the polymer and the QDs themselves, reducing photoluminescence efficiency and spectral stability [27,28]. In addition, the rapid curing process can lead to internal stresses, non-uniform QD dispersion, and shrinkage-induced cracks [29]. These effects are particularly problematic when scaling to large-area displays or achieving high pixel density in high-resolution displays. Moreover, some UV-curable resins absorb part of the blue light emitted by the OLED, decreasing the excitation efficiency of QDs and thus lowering the conversion efficiency [30].

In this paper, we investigated a color conversion layer in which QDs were dispersed in a thermally curable polydimethylsiloxane (PDMS). PDMS is a silicon-based elastomer known for its high optical transparency in the visible and near UV range, excellent thermal and environmental stability, and low elastic modulus [31]. Unlike UV-cured systems, PDMS curing does not require irradiation and proceeds uniformly through thermal crosslinking, minimizing light-induced degradation and allowing more homogenous QD dispersion [32]. Although several authors have demonstrated color conversion layers fabricated using the nanocomposites composed of QDs dispersed in PDMS, most of the research has focused on color conversion for the white LEDs [33,34,35]. In this paper, we report on a PDMS-based QD color conversion layer for use as a pixel component in QD-OLEDs. By varying the QD concentration and layer thickness, we fabricated PDMS-based QD color conversion layers and investigated their color conversion characteristics, including color conversion spectrum, conversion efficiency, and blue leakage.

## 2. Materials and Methods

Blue OLEDs were fabricated using ITO-coated glass substrates with a sheet resistance of about 10 Ω/sq. ITO anode patterns were defined using a photolithography process. The patterned ITO substrates were cleaned with acetone, isopropyl alcohol, and deionized water, followed by oxygen plasma treatment to remove organic residues before evaporating the organic materials. Organic and metal layers were deposited by the vacuum thermal evaporation method at a base pressure of 10^−6^ Torr. After depositing a 50 nm thick N,N′-bis(naphthalen-1-yl)-N,N′-bis(phenyl)-benzidine (NPB) hole transport layer, a 50 nm thick 4,4′-bis(2,2-diphenylethenyl)-1,1′-biphenyl (DPVBi) luminescent layer was evaporated. Finally, 0.5 nm thick LiF and a 100 nm thick Al layer were sequentially evaporated to define the cathodes through a shadow mask without breaking the vacuum. The completed device structure is ITO/NPB (50 nm)/DPVBi (50 nm)/LiF (0.5 nm)/Al. The devices were encapsulated in a nitrogen glove box. The electroluminescence (EL) spectra of the devices were measured using a spectroradiometer (CS1000, Minolta, Tokyo, Japan). To ensure measurement accuracy, the spectroradiometer was periodically calibrated using a standard luminance source traceable to national standards by the supplier. The spectroradiometer was warmed up for at least 30 min prior to data acquisition. Dark calibration was performed prior to each measurement to eliminate the influence of dark current and electronic noise. All measurements were conducted in a light-tight dark box under fixed optical geometry to minimize ambient light and systematic errors.

Green-emitting CdSe/ZnS QDs (ZEUS, Hwaseong, Republic of Korea) with oleic acid ligands were used for fabricating the PDMS-based QD color conversion layers. PDMS and curing agent were purchased from Sewang Hitech Silicone (Kimpo, Republic of Korea). To ensure uniform dispersion, the QDs were first dispersed in octane, which is a nonpolar solvent suitable for stabilizing ligand-capped QDs. Four different QD concentrations of 100, 200, 400, and 800 mg were each dissolved in 1 mL of octane and stirred at 1500 rpm for over 3 h. The QD-octane solution was then mixed with 1 g of PDMS base and 0.1 g of a thermal curing agent, followed by stirring at 300 rpm for 30 min. The resulting PDMS-QD solution was spin-coated and cured on a hot plate at 150 °C for 30 min to form the QD color conversion layers. The fabricated color conversion layer was placed on top of a blue OLED, and its conversion spectrum was measured using a spectroradiometer. The layer thickness was measured using a surface profilometer (Surfcorder ET3000, Kosaka Laboratory, Tokyo, Japan). The shape and size distributions of the QDs were investigated by transmission electron microscopy (TEM, JEM-ARM200F, JEOL, Tokyo, Japan). Time-resolved photoluminescence (TRPL) was measured using a modular research fluorometer for lifetime and steady state measurements (HORIBA, Fluorolog-QM, Kyoto, Japan)

## 3. Results and Discussion

**Figure 1:**
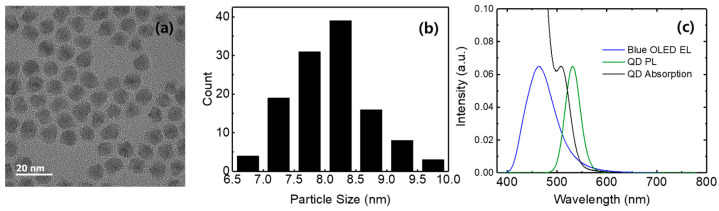
(**a**) TEM image, (**b**) particle size distribution, and (**c**) PL and absorption spectra of the green-emitting CdSe/ZnS QDs, and EL spectrum of the blue OLED.

Figure 1 shows the TEM image, particle size distribution, photoluminescence (PL), and absorption spectra of the green-emitting CdSe/ZnS QDs, as well as the EL spectrum of the blue OLED. The QDs exhibit nearly spherical shapes. The particle sizes of QDs range from 6.5 to 10.0 nm, with the highest population in the range of 8.0–8.5 nm. The average particle size was measured to be 8.1 nm. The PL spectrum of QDs dispersed in octane exhibits an emission peak at 534 nm with a full width half maximum (FWHM) of the spectrum was 45 nm. The blue OLED with a structure of ITO/NPB/DPVBi/LiF/Al exhibits an EL emission peak at 464 nm with a FWHM of 68 nm. In addition, the emission spectrum of the blue OLED significantly overlaps with the absorption region of the QDs, enabling efficient excitation.

**Figure 2:**
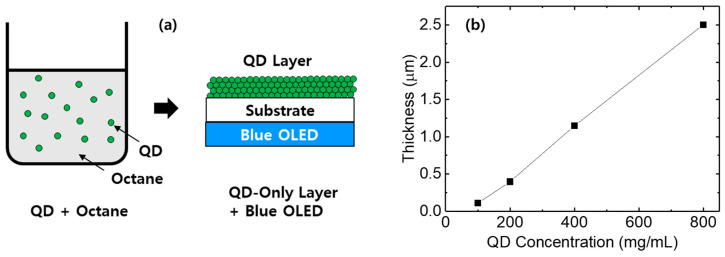
(**a**) Schematic illustration of the fabrication of QD-only layer for color conversion of the blue OLED and (**b**) thickness of the QD-only layer as a function of QD concentration.

To investigate the effect of the PDMS matrix on color conversion, the color conversion characteristics of a QD-only layer were first examined. A QD solution was prepared by dispersing QDs in octane, and a QD-only layer was formed via spin coating. Figure 2a shows a schematic illustration of the QD layer fabrication process and the color conversion process using the blue OLED. The concentration of QDs dispersed in octane was varied to 100, 200, 400, and 800 mg/mL. Figure 2b shows the thickness of the spin-coated QD-only layers as a function of QD concentration. As the QD concentration increases, more QDs accumulate after spin coating, resulting in an increase in the layer thickness.

**Figure 3:**
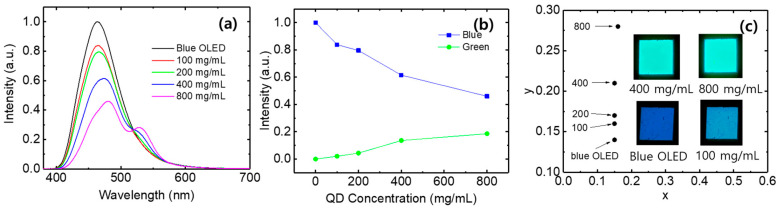
(**a**) Color conversion spectra, (**b**) blue and green emission intensities, (**c**) color coordinates and emission images of QD-only layers fabricated with different QD concentrations.

Figure 3a shows the color conversion spectra of QD-only layers prepared with different concentrations of QDs. The color conversion of the blue OLED by the QDs occurs as the QDs absorb the light emitted from the blue OLED, and the absorption of blue light depends on the thickness of the QD layer. When the QD concentration is 100 mg/mL, the QD layer is too thin, resulting in almost no color conversion. On the other hand, a clearly distinguished green peak from the QDs is observed when the QD concentration increases to 800 mg/mL. In addition, the peak wavelength of the transmitted blue light moves toward the longer wavelength region as the QD concentration increases. For example, the emission peak wavelength of the blue OLED is 464 nm, whereas the QD conversion layer at 800 mg/mL exhibits a peak wavelength of 480 nm. This red shift is considered to be due to the wavelength-dependent absorption characteristics of the QDs. Since QDs exhibit stronger absorption at shorter wavelengths within the blue emission range of the OLED, the higher energy components of the blue emission are preferentially absorbed as the QD layer becomes thicker, while the lower components are relatively less attenuated. This difference in absorption leads to an apparent red shift.

Figure 3b shows the maximum intensities of the transmitted blue light and the converted green light as a function of QD concentration. As the QD concentration increases, the thickness of the QD layer increases, resulting in greater absorption of blue light by the QDs. Consequently, the intensity of the converted green light increases, while the intensity of the transmitted blue light decreases. Figure 3c shows the color coordinates and emission images of the light converted by the QD-only layers. The blue OLED exhibits color coordinates of (0.15, 0.14). At a QD concentration of 100 mg/mL, little color conversion occurs, and the color coordinates are nearly identical to those of the blue OLED, at (0.15, 0.16). As the QD concentration increases, more blue light is absorbed by the QDs, causing the color coordinates to shift toward the green region. At a QD concentration of 800 mg/mL, the color coordinates are (0.16, 0.28). Although the color coordinates shift toward the green region, the blue emission remains dominant and the green emission is relatively weak, resulting in a greenish-blue color. In addition, the color-converted images become increasingly blurred and diffused with increasing QD concentration, indicating enhanced light scattering within the layer.

**Figure 4:**
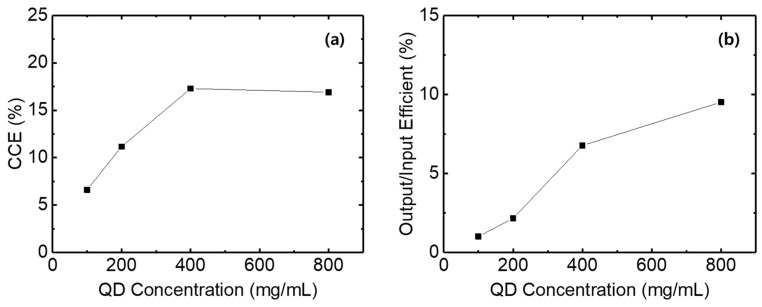
(**a**) Color conversion efficiencies and (**b**) output/input efficiencies of QD-only layers fabricated with different QD concentrations.

Figure 4 shows the color conversion efficiencies and the output/input efficiencies of the QD-only layer as a function of QD concentration. The color conversion efficiency is defined as follows [24]: η_CCE_ = A_QD_/(A_bOLED_ − A_btrans_) × 100, where η_CCE_ is the color conversion efficiency, A_QD_ is the integrated area of the converted green emission spectrum, A_bOLED_ is the integrated area of the blue light spectrum incident from the OLED onto the QD layer, and A_btrans_ is the integrated area of the blue light spectrum transmitted through the QD layer. The output/input efficiency is defined as follows [24]: η_out/in_ = A_QD_/A_bOLED_ × 100, where η_out/in_ is the output/input efficiency. A_QD_ and A_bOLED_ are the same as defined above.

The QD-only layer exhibits a low color conversion efficiency of 6.6% due to the thin thickness at a QD concentration of 100 mg/mL. As the QD concentration increases to 400 mg/mL, the thickness of the QD-only layer increases, and the color conversion efficiency rises to 17.3%. When the QD concentration reaches 800 mg/mL, the color conversion efficiency slightly decreases to 16.9%. In contrast, the output/input efficiency exhibits lower values than the color conversion efficiency, because it does not account for the transmitted blue light, i.e., blue leakage. At a QD concentration of 100 mg/mL, about 85% of the blue light is leaked without being absorbed by the QDs, resulting in a very low proportion of converted light, approximately 1%. As the QD concentration increases, a higher proportion of blue light is absorbed by the QDs. Consequently, the output/input efficiency increases, reaching 9.5% at a QD concentration of 800 mg/mL.

**Figure 5:**
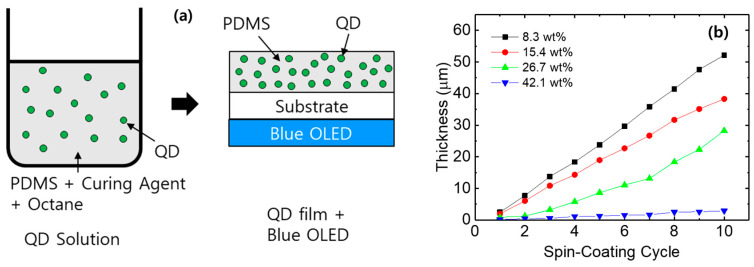
(**a**) Schematic illustration of the fabrication of the PDMS-based QD color conversion layer, and (**b**) thickness of the PDMS-based color conversion layer as a function of spin-coating cycle.

To fabricate the color conversion layers by dispersing QDs in PDMS, QDs were first added to octane to prepare a QD solution, which was then mixed with PDMS containing a curing agent. Figure 5a shows a schematic illustration of the fabrication process of the color conversion layer in which QDs are dispersed in PDMS, as well as a schematic of the color conversion process using a blue OLED. For the color conversion layers, 1 g of PDMS and 0.1 g of curing agent were used. In addition, the concentration of QDs in 1 mL of octane was varied to 100, 200, 400, and 800 mg/mL. Therefore, the concentrations of QDs dispersed in PDMS were 8.3, 15.4, 26.7, and 42.1 wt%, respectively. After spin-coating the solution containing PDMS, curing agent, octane, and QDs, the coated films were cured at 150 °C for 30 min to form the color conversion layers. The thickness of the color conversion layers was controlled by repeating the spin-coating and curing processes.

Figure 5b shows the thickness of the color conversion layers as a function of the number of repeated spin-coating and curing cycles. The thickness of the layer increases as the number of spin-coating cycles increases, as the layer cured at 150 °C remains undamaged when subsequent PDMS-QD mixtures are spin-coated onto it. It should be noted that the thickness of the color conversion layer decreases with increasing QD concentration despite the higher solid content. The decrease in the thickness of the color conversion layer at higher QD concentrations is presumed to be related to the plasticizing effect of oleic acid ligands coordinated on the QDs [36]. As the QD concentration increases, the amount of oleic acid also increases, which is believed to reduce the viscosity and surface tension of the PDMS-QD solution [36,37]. The QD ligands can also delay the PDMS curing kinetics, allowing the wet film to flow for a longer time during spin-coating, which can result in the formation of a thinner layer [38]. We observed a noticeable increase in the fluidity of the PDMS-QD mixture with high QD loading. This reduced viscosity allows the solution to spread more thinly during the spin coating process, resulting in a decrease in the final film thickness. On the other hand, we observed that when the QD concentration exceeded 42.1 wt%, it was impossible to measure the thickness of the layer using a surface profiler because curing of the layer did not occur. This result suggests that an excessive amount of oleic acid ligands hinders the network formation of the PDMS matrix.

**Figure 6:**
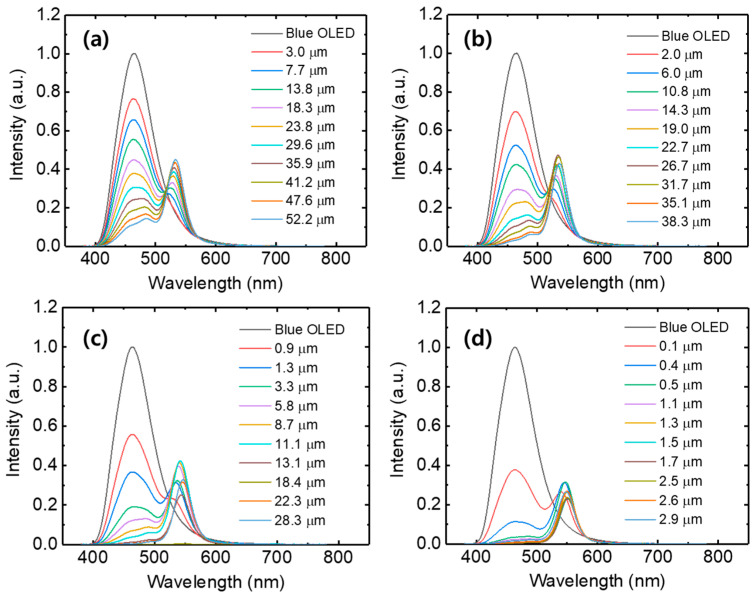
Color conversion spectra of PDMS-based QD color conversion layers with QD concentrations of (**a**) 8.3 wt%, (**b**) 15.4 wt%, (**c**) 26.7 wt%, and (**d**) 42.1 wt%.

Figure 6 shows the color conversion spectra of the PDMS-based QD color conversion layers. As the layer thickness increases, the transmitted blue emission decreases and the converted green emission increases for all QD concentrations. At a low concentration of 8.3 wt%, the blue emission dominates due to the limited absorption of the incident blue light. As the QD concentration increases to 15.4 wt% and 26.7 wt%, the QD emission progressively increases, while the blue leakage correspondingly decreases. At the highest concentration of 42.1 wt%, the blue emission is significantly suppressed, and the QD emission becomes dominant. These results indicate that the PDMS-based QD color conversion layers exhibit significantly lower blue leakage and improved color conversion characteristics compared with the QD-only layers.

**Figure 7:**
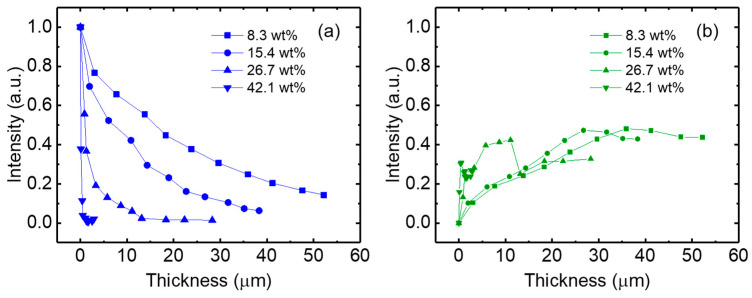
(**a**) blue and (**b**) green emission intensities of PDMS-based QD color conversion layers with different QD concentrations.

Figure 7 shows the variation in the maximum intensities of transmitted blue light and converted green light for PDMS-based QD color conversion layers with different QD concentrations. As the thickness increases, the blue intensity decreases for all QD concentrations, indicating the enhanced absorption of the incident blue light within the QD conversion layer. This trend becomes more pronounced at higher QD concentrations, where the stronger absorption leads to a more rapid attenuation of the blue emission with increasing thickness. The corresponding green emission intensity increases with increasing layer thickness due to the enhanced color conversion by the QDs. However, beyond a certain thickness, the green emission intensity begins to saturate or slightly decreases. In addition, at higher QD concentrations, the green emission reaches its maximum at a relatively smaller thickness.

**Figure 8:**
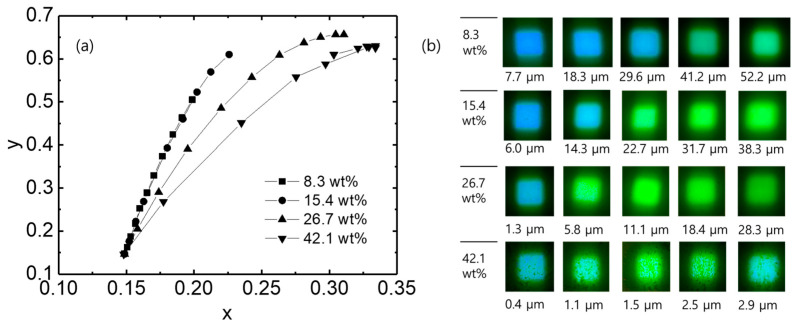
(**a**) Color coordinates and (**b**) color conversion images of PDMS-based QD color conversion layers with different QD concentrations.

Figure 8a shows the color coordinates of the PDMS-based QD color conversion layers with different concentrations of QDs. As the layer thickness and the QD concentration increase, the emission color systematically shifts from the blue region toward the green region. This result directly reflects the progressive suppression of the blue leakage and the enhancement of the green emission. For example, the color coordinates are (0.23, 0.61) in the 25.1 µm layer with 15.4 wt% QDs. Therefore, the color coordinates of the PDMS-based QD layer are significantly improved compared to those of the QD-only layer and are closer to the standard green coordinates (0.19, 0.80) of Rec. 2020. Figure 8b shows the corresponding color conversion images. As the QD concentration and thickness increase, the emission color gradually shifts from bluish green to a more saturated green. Figure 8b also represents that the color-converted images become increasingly blurred and diffused with increasing QD concentration and layer thickness, indicating enhanced light scattering within the PDMS-based QD layer. This suggests that the QDs act as scattering centers in the PDMS matrix, inducing multiple scattering of blue light. This scattering increases the effective optical path length of blue photons within the layer. This light-trapping effect enhances the probability of blue light absorption by the QDs. Consequently, blue leakage is significantly suppressed, and the green emission is correspondingly improved. However, spatial inhomogeneities can be observed in the color conversion images of 42.1 wt% QDs, which may be attributed to QD aggregation within the PDMS matrix. This aggregation results in non-uniform emission and slight degradation of color purity.

**Figure 9:**
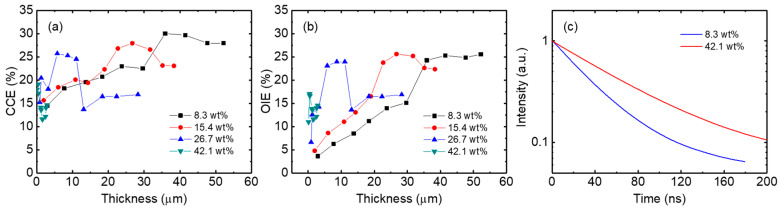
(**a**) Color conversion efficiencies, (**b**) output/input efficiencies of PDMS-based QD color conversion layers with different QD concentrations, and (**c**) TRPL decay curves of PDMS-QD solutions.

Figure 9 shows the color conversion efficiencies and output/input efficiencies of PDMS-based QD color conversion layers, and TRPL decay curves of the PDMS-QD solutions. The color conversion efficiency increases with increasing thickness, reaches a maximum value, and then slightly decreases for all QD concentrations. The 8.3 wt% layer exhibits a maximum color conversion efficiency of 30.0% at a thickness of 35.9 µm, while the 15.4 wt% and 26.7 wt% layers show maximum color conversion efficiencies of 26.7% and 25.7% at thicknesses of 27.9 and 5.8 µm, respectively. The 42.1 wt% layer exhibits a maximum color conversion efficiency of 19.0% at a thickness of 0.4 µm. This result indicates that the optimal thickness for maximum color conversion efficiency decreases with increasing QD concentration. Furthermore, the maximum color conversion efficiency also decreases as the QD concentration increases. A similar trend is observed in the output/input efficiency, where both the maximum output/input efficiency and corresponding optimal thickness decrease with increasing QD concentration.

To further investigate these efficiency characteristics, TRPL measurements of the PDMS-QD solutions were performed. The PL decay is significantly prolonged as the QD concentration increases from 8.3 wt% to 42.1 wt%, indicating enhanced self-absorption in the dense QD environment. In such a high-density QD system, photons emitted by the QDs are likely to be reabsorbed by neighboring QDs before escaping the sample, thereby extending the measured decay time [39]. These results suggest that the PDMS-QD system effectively traps and recycles photons through multiple scattering and reabsorption events. In the PDMS-QD layer, multiple scattering increases the effective optical path length, thereby enhancing the probability of blue photon absorption and improving color conversion efficiency while suppressing blue leakage compared to QD-only films. However, at higher QD concentrations, excessive self-absorption leads to repeated photon recycling processes, during which photons are increasingly lost via non-radiative recombination pathways. In addition, serious QD agglomeration in the 42.1 wt% layers may further exacerbate self-absorption and reduce the maximum color conversion efficiency.

## 4. Conclusions

Color conversion layers were fabricated by dispersing QDs in a thermally curable PDMS matrix, and the color conversion characteristics of blue light emitted from a blue OLED were compared with those of QD-only layers. QD-only layers were prepared by spin-coating a QD solution dispersed in octane. As the QD concentration in octane increased from 100 to 800 mg/mL, the thickness of the QD-only layer increased from 0.11 to 2.51 µm, resulting in enhanced absorption of blue light by the QDs. Consequently, the green emission increased while the blue leakage decreased. However, a considerable amount of blue light was transmitted through the layer, resulting in a relatively low maximum color conversion efficiency of 17.3%. In contrast, the PDMS-based QD color conversion layer exhibited significantly improved color conversion characteristics. PDMS-based layers were fabricated by spin-coating a mixture of thermally curable PDMS, a curing agent, octane, and QDs. The concentration of QDs varied from 8.3 to 42.1 wt%, and the thickness of the QD layer was controlled by repeating the spin-coating and thermal curing processes. The PDMS-based QD layers exhibited significantly reduced blue leakage and substantially increased green emission intensity compared with the QD-only layers. At a QD concentration of 8.3 wt% and a layer thickness of 35.9 µm, the PDMS-based layer exhibited a maximum color conversion efficiency of 30.0%. Higher QD concentrations slightly reduced the maximum efficiency due to the photon loss caused by excessive self-absorption, although blue leakage was suppressed even with thinner layers. These results indicate that the PDMS-based QD color conversion layer provides improved blue light absorption, reduced blue leakage, and enhanced color conversion efficiency, demonstrating its strong potential for high-performance QD-OLED display applications.

## Data Availability

The original contributions presented in this study are included in the article. Further inquiries can be directed to the corresponding author.
